# Metagenomic and Biochemical Characterizations of Sulfur Oxidation Metabolism in Uncultured Large Sausage-Shaped Bacterium in Hot Spring Microbial Mats

**DOI:** 10.1371/journal.pone.0049793

**Published:** 2012-11-21

**Authors:** Satoshi Tamazawa, Kazuto Takasaki, Hideyuki Tamaki, Yoichi Kamagata, Satoshi Hanada

**Affiliations:** 1 Bioproduction Research Institute, National Institute of Advanced Industrial Science and Technology (AIST), Tsukuba, Ibaraki, Japan; 2 Graduate School of Life and Environmental Sciences, University of Tsukuba, Tsukuba, Ibaraki, Japan; 3 Division of Applied Bioscience, Graduate School of Agriculture, Hokkaido University, Sapporo, Japan; University of Delaware, United States of America

## Abstract

So-called “sulfur-turf” microbial mats in sulfide containing hot springs (55–70°C, pH 7.3–8.3) in Japan were dominated by a large sausage-shaped bacterium (LSSB) that is closely related to the genus *Sulfurihydrogenibium*. Several previous reports proposed that the LSSB would be involved in sulfide oxidation in hot spring. However, the LSSB has not been isolated yet, thus there has been no clear evidence showing whether it possesses any genes and enzymes responsible for sulfide oxidation. To verify this, we investigated sulfide oxidation potential in the LSSB using a metagenomic approach and subsequent biochemical analysis. Genome fragments of the LSSB (a total of 3.7 Mb sequence including overlapping fragments) were obtained from the metagenomic fosmid library constructed from genomic DNA of the sulfur-turf mats. The sequence annotation clearly revealed that the LSSB possesses sulfur oxidation-related genes coding sulfide dehydrogenase (SD), sulfide-quinone reductase and sulfite dehydrogenase. The gene encoding SD, the key enzyme for sulfide oxidation, was successfully cloned and heterologously expressed in *Escherichia coli*. The purified recombinant enzyme clearly showed SD activity with optimum temperature and pH of 60°C and 8.0, respectively, which were consistent with the environmental conditions in the hot spring where the sulfur-turf thrives. Furthermore, the affinity of SD to sulfide was relatively high, which also reflected the environment where the sulfide could be continuously supplied. This is the first report showing that the LSSB harbors sulfide oxidizing metabolism adapted to the hot spring environment and can be involved in sulfide oxidation in the sulfur-turf microbial mats.

## Introduction

A wide variety of microbial mats develop in geothermal hot springs, and form the unique microbial ecosystems composed of physiologically and phylogenetically diverse microbes. Sulfide is dissolved in geothermal waters of various hot springs all over the world, and considered as one of the important energy sources in those particular ecosystems. Indeed, sulfide-containing hot springs form colorful microbial mats (white, gray, yellow, red, pink, green, etc.) harboring different types of microbes (e.g., lithotrophic sulfur oxidizing bacteria, anoxygenic phototrophic bacteria, cyanobacteria and heterotrophic bacteria depending these autotrophs) [1]–[8]. Although a few main constituents in those mats were previously isolated [9], [10], the majority of them are yet to be cultured and their physiological and ecological functions have not been fully understood.

White microbial mats, so-called sulfur-turf, develop in high-temperature sulfidic hot springs [2], [6]. The sulfur-turf mats are dominated by an uncultured large sausage-shaped bacterium (LSSB) with a large amount of elemental sulfur particles around its cells. Phylogenetic analysis based on 16S rRNA gene revealed that the LSSB was related to the genus *Sulfurihydrogenibium* within the phylum *Aquificae* [11], [12]. To date, five species and the remaining isolates of *Sulfurihydrogenibium*, e.g., *Sulfurihydrogenibium subterraneum*, *S. azorense*, *S. yellowstonense*, *S. rodmanii* and *S. kristjanssonii,* have been isolated and validly described [13]–[17]. The first species was isolated from a subsurface hot aquifer water in a gold mine in Japan [13], [18], and all the remains were isolated from terrestrial hot springs in Portuguese, the United States (Yellowstone), Russia and Iceland [14]–[17]. All of the isolates show rod-shaped cells with less than 2.8 µm long and are thermophilic, microaerophilic, elemental sulfur- and thiosulfate-utilizing chemolithoautotrophic organisms. By contrast, the LSSB is, as the name implies, sausage shaped and gigantic bacterium with length of 5–20 µm. This conspicuous organism is widespread in sulfide-containing hot springs. Some previous studies proposed that the uncultured LSSB could be associated with sulfide oxidation in the sulfur-turf microbial mats in the hot springs. Maki (1987) reported that the sulfur-turf mats oxidize sulfide to elemental sulfur, and further oxidize the precipitated elemental sulfur to sulfate via thiosulfate [19]. Nakagawa and Fukui (2003) suggested that there was an active sulfur cycle between the LSSB as a sulfide consumer and *Thermodesulfobacteria*-like microbes as a sulfide producer in sulfide-containing hot spring streamers [11]. Recently, Kubo et al. (2011) also hypothesized that LSSB might be a sulfide oxidizer in the sulfidic hot spring [12]. However, no report shows that any known *Sulfurihydrogenibium* isolates closely related to the LSSB can utilize sulfide, although they are known to oxidize elemental sulfur and thiosulfate. Furthermore, there is no direct evidence in genetic and biochemical respects showing that LSSB possesses sulfide oxidation metabolism so far due to the lack of its genomic information as well as the axenic culture.

**Figure 1:**
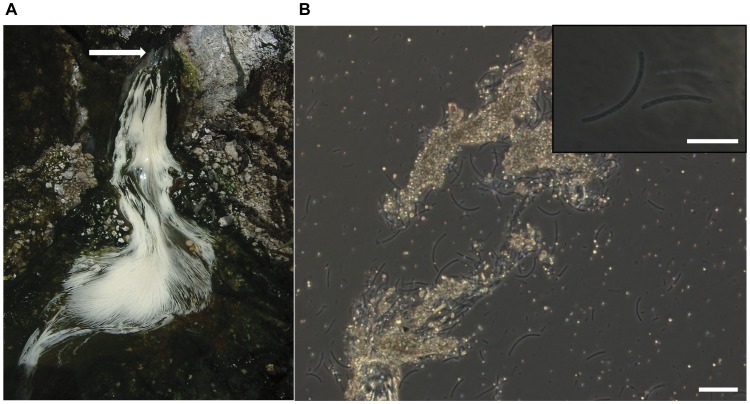
Photographs of sulfur-turf microbial mat. [A] A photograph of sulfur-turf microbial mat found in the hot spring stream, [B] a phase contrast photomicrograph of the LSSB with elemental sulfur granules. An arrow indicates flowing channel of sulfide-containing hot spring water. Scale bar = 10 µm.

To clarify the sulfide-oxidizing metabolism and its related functions in the LSSB, we conducted metagenomic and biochemical investigations of the sulfur-turf mats in the sulfide rich hot spring in Japan in this study. In particular, we constructed metagenomic fosmid libraries from genomic DNA of the sulfur-turf mats in which LSSB dominated, and annotated the key genes responsible for sulfide oxidation metabolism in the LSSB genome fragment obtained. Furthermore, based on the metagenomic information, we succeeded in cloning, heterologous expression, and biochemical characterization of the sulfide dehydrogenase, a critical enzyme for sulfide oxidation.

## Materials and Methods

### Sampling

Sulfur-turf microbial mats were collected from Nakabusa hot spring (55–70°C, pH 7.3–8.3) in Nagano Prefecture, Japan (36°23.483N, 137°44.883E). The microbial mats were taken in sterile plastic tube (50 ml) with spring water, and were immediately placed under anaerobic conditions using the Anaero-Pack system (Mitsubishi Gas Chemical Co., Japan). All necessary permits were obtained for the described field studies by the owner of Nakabusa hot spring.

The samples containing the large sausage-shaped bacterium (LSSB) and glittering elemental sulfur particles were harvested by centrifugation at 3,000 g for 5 min, and washed twice with 50 mM phosphate buffer (pH 7.0) to wash off other contaminated microbes. The washed samples were examined under a phase contrast microscope (OLYMPUS AX80, OLYMPUS, Japan) with a CCD camera (C4742-95-12ER, HAMAMATSU, Japan). The samples were stored at −80°C until use.

### Total DNA Extraction and Purification

The mat sample was freeze-dried by FLEXI-DRY (FTS systems, NY, USA) and grinded by a sterile mortar and pestle. Total genomic DNA was extracted from the freeze-dried sample using direct lysis method described previously [6], [20], purified with chloroform-phenol (1∶1, vol/vol) twice, and washed once with chloroform. The DNA was precipitated with isopropanol, and washed with 70% ethanol. The purified DNA was analyzed on pulsed field gel electrophoresis (Bio-Rad, CA, USA) to ensure that the DNA is not degraded.

**Figure 2:**
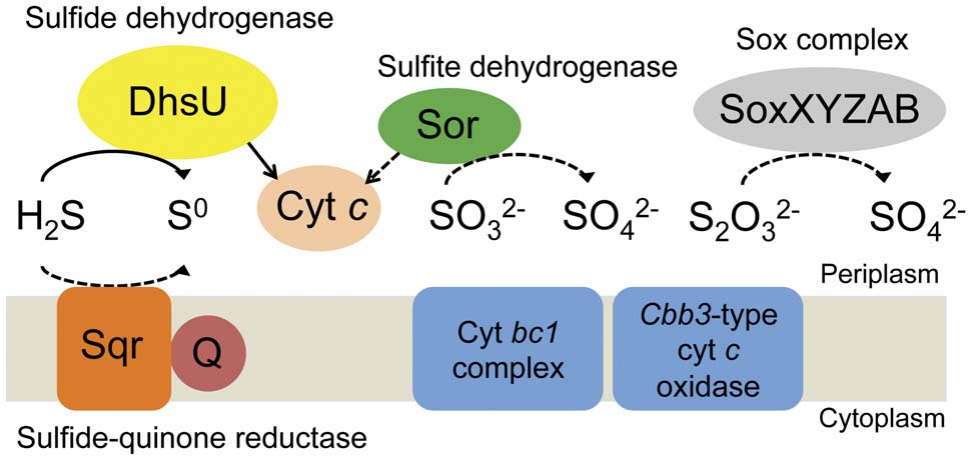
Conceptual model of inorganic sulfur compounds oxidation pathway and respiratory complex in the LSSB. Sulfide dehydrogenase, DhsU; sulfide-quinone reductase, Sqr; sulfite dehydrogenase, Sor; sox complex, SoxXYZAB; quinone, Q; cytochrome *c*, Cyt *c*; cytochrome *bc1* complex, Cyt *bc1* complex; *Cbb3*-type cytochrome *c* oxidase, *Cbb3*-type cyt *c* oxidase. The solid arrow shows the reaction confirmed by both metagenomic and biochemical approaches. The dashed arrows indicate the putative reactions identified by only metagenomic information.

### 16S rRNA Gene Clone Library Analysis

16S rRNA genes were PCR-amplified from the purified DNA by using the bacterial universal primer EUB338 and 1492R as described by Tamaki et al. [21]. The purified rRNA gene fragments were cloned with a pT7Blue T-vector kit (Novagen, Germany). The clonal DNAs were amplified from randomly selected recombinants by direct PCR with M13 primers purified with a QIAquick PCR Purification Kit (QIAGEN, Germany), and then used as templates for sequencing. Sequencing was performed with a universal primer 907R, a DTCS-Quick Start kit (Beckman coulter, CA, USA), and a CEQ-2000 automated sequence analyzer (Beckman coulter). The sequences of all 16S rRNA gene clones (∼660 bp) were determined, compared with those in the GenBank database by using the BLAST program (NCBI-BLAST, www.ncbi.nlm.nih.gov/BLAST), and aligned by ClustalW (ver. 2.1). The sequence similarity of 99% was used as a cutoff value for grouping the sequences into different operational taxonomic units (OTUs).

### Metagenomics

A fosmid library was constructed from the purified DNA that was digested with *Mbo*I (TaKaRa, Japan) using CopyControl pCC1FOS vector system (Epicentre Technologies, WI, USA). Both 3′ and 5′ ends of the 6,432 clones (36 kbp of the average insert size) were sequenced using the BigDyeTerminator Version 3.1 reactions kit (PerkinElmer, MA, USA) v3.1 on ABI3730×l sequencers (Applied Biosystems, CA, USA). The paired-end sequence reads from each fosmid clone were individually assembled to generate non-redundant meta-sequences using the Phred/Phrap/Consed software package (http://www.phrap.org/) [22], [23]. To phylogenetically classify the meta-sequences, XanaMine (Xanagen, Japan), which is based on a self-organizing map (SOM) algorithm, was employed [24]–[26]. To obtain the sequences derived from LSSB, SOM (first SOM) was constructed with tetranucleotide frequencies for 1,720 contigs from the sulfur-turf and 212 complete genome sequences (191 of bacteria and 21 of archaea). The high-dimensional data was approximated and described as the two-dimensional map using XanaMine Viewer (Xanagen). Based on the SOM analysis, the sequences of the LSSB were sorted out as described previously [24]. All the fosmid clones containing the sorted sequences were pooled and randomly sheared to 1–2 kb fragments using a HydroShear (GeneMachines, CA, USA), and were sequenced with the whole-genome shotgun method using ABI3730×l sequencers (Applied Biosystems). These shotgun sequencing reads were assembled together using the Phred/Phrap/Consed software, which produced 3,349 contigs and 10,298 singlets. To screen the sequences of LSSB from *Escherichia coli* and minor contaminated sequences, SOM (second SOM) was reconstructed with tetranucleotide frequencies for the obtained 3,349 contigs and 10,298 singlets and 212 complete genome sequences. Based on the SOM analysis, 3.7 Mbp (1,504 contigs and 135 singlets) of sequences were eventually obtained from the LSSB genome. XanaGenome (Xanagen), a comprehensive database system for microbial genome analysis, was used for automatic and manual annotations of open reading frames (ORFs) that are likely to encode proteins for the screened sequences. NCBI-BLAST for homology search and protein domain analysis via UniProt Knowledgebase (http://ca.expasy.org/) [27] were also conducted for the sequence annotation.

**Figure 3:**
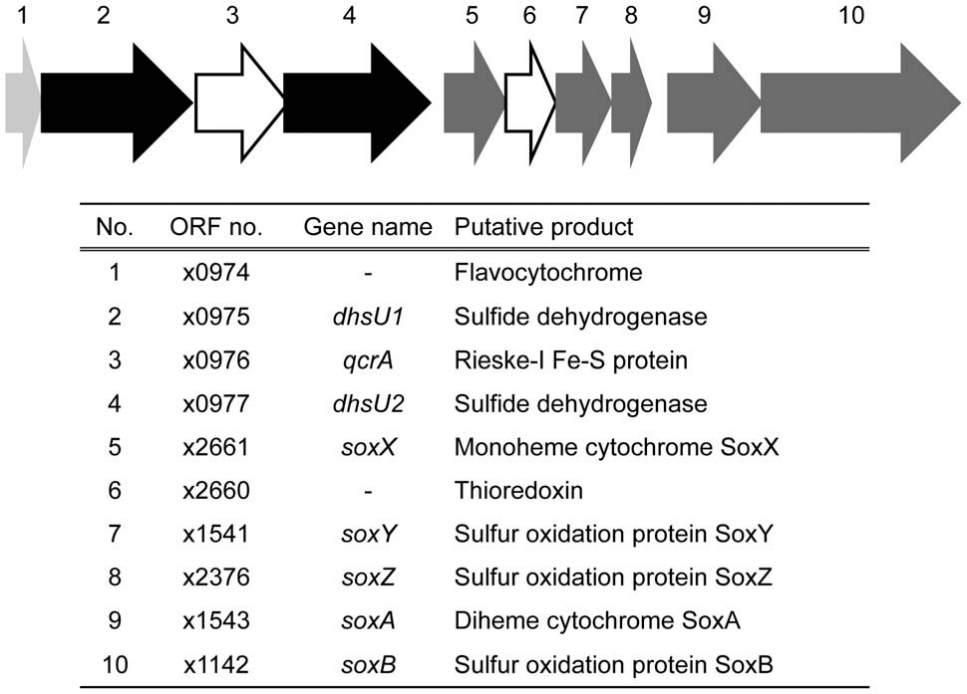
Schematic map of the *dhsU* gene cluster found in the LSSB. Arrows indicate the position and direction of each ORF. Black, light-gray, dark-gray, and white arrows show *dhsU* genes, cytochrome-like genes, sox-related genes, and a Fe-S protein gene and a ferredoxin gene, respectively.

### Amino Acid Sequence Analysis of the SD Gene (*dhsU1*)

ClustalW (ver. 2.1) was used for multiple alignments of amino acid sequences of sulfide dehydrogenase (SD) and putative SD. Phylogenetic tree of SDs was constructed by a neighbor-joining method based on the aligned amino acid sequence data. Signal sequence of SD (DhsU1) was predicted by SignalP 3.0 (http://www.cbs.dtu.dk/services/SignalP/).

### Cloning of the SD Gene (*dhsU1*)

Based on the metagenome information, a primer set specific to the *dhsU1* gene of the LSSB was designed. The forward primer (dhsU1F2B, 5′-GGATCCGGCCATGTCTTCAAAA-3′) and the reverse primer (dhsU1R2X, 5′-GTTTCTCGAGTCCCTTACCCCAT-3′) contain a *Bam*HI restriction site (underlined) and a *Xho*I restriction site (underlined), respectively. The PCR-amplification of the *dhsU1* gene was performed as follows: an initial denaturation step at 95°C for 2 min, followed by 30 cycles of denaturation at 95°C for 30 s, annealing at 52°C for 30 s, extension at 72°C for 1.5 min, and a final extension step at 72°C for 5 min. The PCR products were cloned with a pT7Blue T-Vector Kit. The clonal DNAs were excised with *Bam*HI and *Xho*I, and re-ligated into the pET-28b Vector (Novagen) digested with the same restriction enzymes (pT28dhsU1).

**Table 1:** Purification of recombinant DhsU1 of the LSSB.

	Total protein (mg)	Total activity (U)	Specific activity (U/mg)	Purification (fold)	Yield (%)
Crude extract	187.6	5.774	0.031	1.0	100
Ni-NTA	9.2	2.344	0.255	8.3	40.6

The reaction mixture contained 100 mM Tris-HCl (pH 8.0), 30 µM horse heart cytochrome *c*, 10 µM sodium sulfide and 25 µg crude or purified DhsU1. The total volume of the reaction mixture was 1.0 ml. The reaction was started by adding sodium sulfide. One unit of activity (U) is defined as 1 µmol of horse-heart cytochrome *c* reduced per minute.

### Expression and Purification of the Recombinant SD (DhsU1)

The pT28dhsU1 was transformed into *E. coli* BL21(DE3) (TaKaRa). The recombinant *E. coli* was cultured at 37°C with shaking in LB liquid medium supplemented with kanamycin (30 µg/ml) until OD_600_ reached 0.5–1.0 and then induced with 0.5 mM isopropyl β-D-1-thiogalactopyranoside (IPTG) at 25°C for 16 h. The cultured cells were harvested by centrifugation at 8,000 g for 10 min at 4°C. The cells were frozen and thawed once, and resuspended in lysis buffer containing 100 mM Tris-HCl (pH 8.0), 250 mM NaCl, and 0.05% Tween 20. The lysate was disrupted by ultra sonication on ice, and then the crude extracts were collected by centrifugation at 15,000 g for 20 min at 4°C. The crude extracts were purified using His-Select Nickel Affinity Gel (Sigma, MO, USA) according to the manufacturer’s instructions. Eluted fractions were equilibrated with 100 mM Tris-HCl (pH 8.0) at least twice using Spectra/Por Dialysis Membrane MW 3500 (Spectrum Laboratories, CA, USA), and were finally concentrated using Amicon Ultra-15 (Millipore, Ireland). The protein was quantified by the method of Bradford [28].

**Figure 4:**
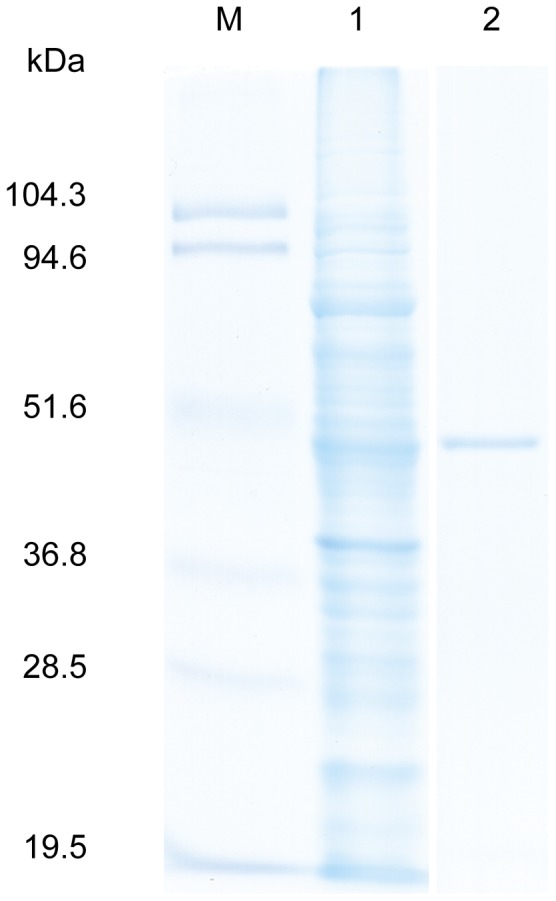
SDS-PAGE of recombinant DhsU1 of the LSSB. Lane 1, crude fraction; lane 2, purified DhsU1; M, molecular weight standard (19.5–104.3 kDa).

### Protein Electrophoresis

The homogeneity and molecular masses of the denatured recombinant proteins were estimated by sodium dodecyl sulfate polyacrylamide gel electrophoresis (SDS-PAGE) using 10% polyacrylamide gel [29]. The gel was stained with Bio-Safe CBB G-250 (BioRad) after electrophoresis.

### UV/visible Spectroscopy

Ultra-violet/visible spectra of the purified recombinant enzyme were measured with DU800 SPECTROPHOTOMETER (Beckman coulter) under the following conditions: wavelength Interval; 1.0 nm, Scan Speed; 1200 nm/min, wavelength range; 300 nm–800 nm. The reduced spectra were immediately detected after addition of a trace amount of dithionite.

**Figure 5:**
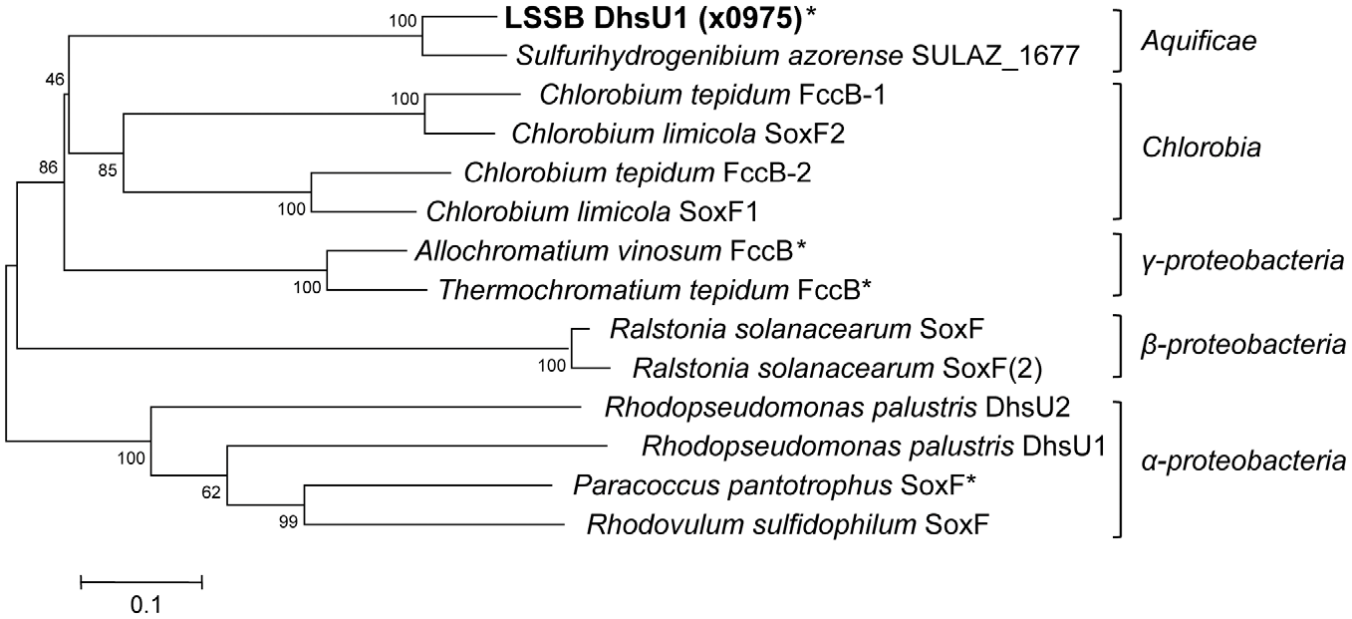
Phylogenetic relationship of DhsU1 homologs including real and putative SD between LSSB and other bacteria. The tree was constructed using neighbour-joining method based on the amino acid sequences. The scale bar indicates 0.1 substitutions per amino acid position. The numbers at nodes represent bootstrap values (100 replicates). Asterisk indicates the real SDs previously proved to have its enzymatic activity *in vitro*.

### Enzyme Assay

SD activity was determined with the reaction mixture containing 100 mM Tris-HCl (pH8.0), 30 µM horse heart cytochrome *c*, 10 µM disodium sulfide and 50 µg of enzyme. The reaction was performed at 60°C and started by addition of disodium sulfide. The reduction of horse heart cytochrome *c* was detected at 550 nm using DU800 SPECTROPHOTOMETER (Beckman Coulter). Non-enzymatic reduction of horse heart cytochrome *c* was subtracted from the reduction value mentioned above. One unit of enzyme activity is defined as 1 µmol of horse heart cytochrome *c* reduced per minute.

**Figure 6:**
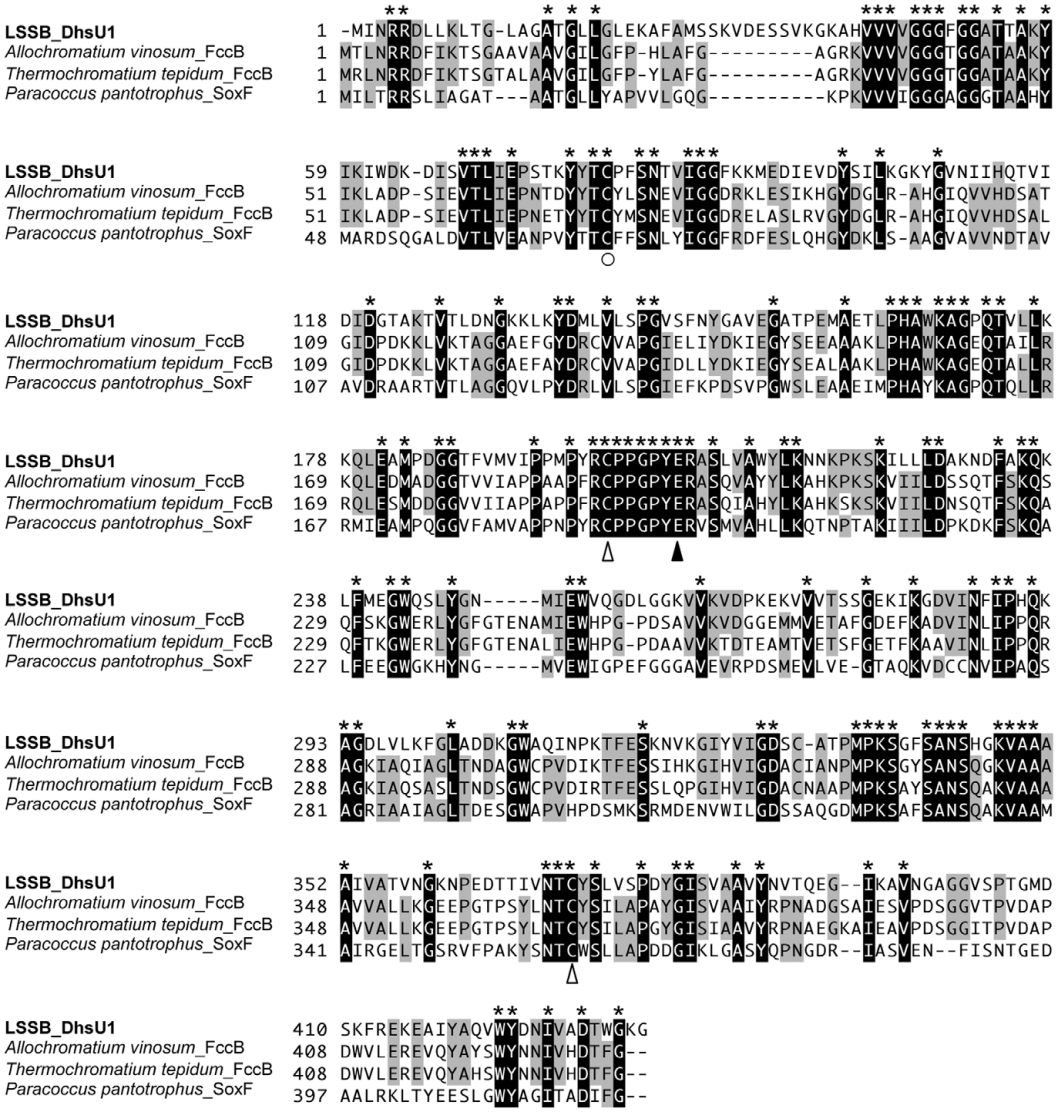
Multiple alignment of SD amino acid sequences of LSSB and other bacteria with its activity. The active site, FAD binding site and disulfide-bond residues are indicated by ○, ▴ and △, respectively.

The optimal temperature of the purified recombinant enzyme was determined in a temperature range of 40–80°C. The optimal pH of the enzyme was also determined from pH 7.0 to 10.0 with following buffers: 50 mM Tris-HCl for pH 7.0 to 9.0; *N*-cyclohexyl-3-aminopropanesulfonic acid (CAPS) for pH 9.5 to 10.0. The thermostability of the enzyme was investigated by measuring the residual activity after pre-incubation of the enzyme at 50, 60, 70 and 80°C for 30 min.

The substrate specificity of the purified recombinant enzyme was determined using different substrates, sulfur (0.02, 0.2 and 2 mM), sulfite (0.02 and 0.4 mM) and thiosulfate (0.02 and 2 mM). SD activity was determined as described above.

For inhibition test, potassium ferricyanide known as a SD inhibitor was used. After pre-incubation of the recombinant enzyme with 10 µM or 25 µM potassium ferricyanide for 5 min at 25°C, the SD activity was measured as described above.

Kinetic properties of the recombinant enzyme were measured at various concentrations of disodium sulfide (2–20 µM) at 60°C in 100 mM Tris-HCl (pH 8.0), 30 µM horse heart cytochrome *c* and 50 µg of enzyme, and at various concentrations of horse heart cytochrome *c* (5–100 µM) at 60°C in 100 mM Tris-HCl (pH 8.0), 10 µM disodium sulfide and 50 µg of enzyme.

**Table 2:** Comparisons of kinetic properties of recombinant DhsU1 of the LSSB with previously known SDs from other bacteria.

	This study	*P. pantotrophus*	*Thiobacillus* sp. W5
*K*_m_ for sulfide (µM)	16.5	2.3	1.7
*K*_m_ for horse-heart cytochrome *c* (µM)	52.0	116	3.8
*V*_max_ (µmol mg^−1^ min^−1^)	2.5	ND*	2.1
*K*_cat_ (s^−1^)	1.9	3.9	1.8

* ND, not determined.

**Figure 7:**
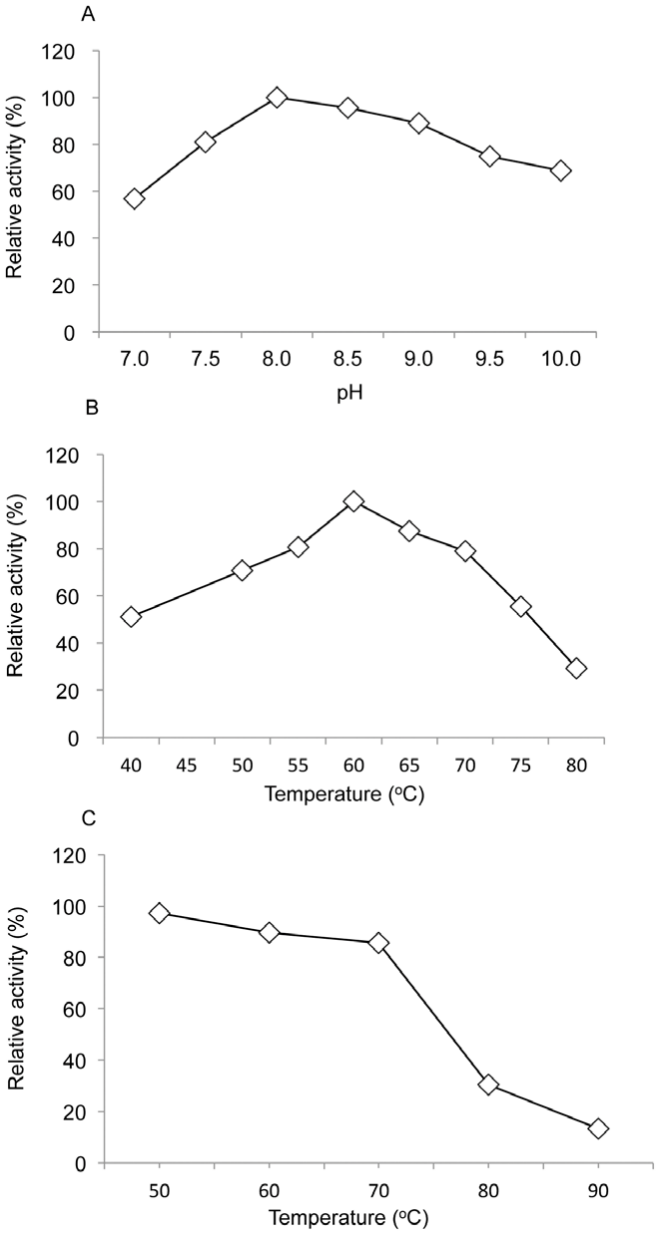
Effect of [A] pH and [B] temperature on recombinant LSSB DhsU1 and [C] its thermostability. The optimal temperature was determined by measuring the enzyme activity at selected temperatures from 40–80°C. The optimal pH was determined in several buffers with a range from 7.0–10.0 (50 mM): Tris-HCl (7.0–9.0) and CAPS (9.5–10.0). The thermostability was determined by using pre-incubated DhsU1 for 30 min at 30–90°C.

### Accession Numbers

The sequences obtained in this study have been deposited in DDBJ with accession number AB735049 to AB735181.

## Results

### Microbial Community of the Sulfur-turf Microbial Mat

Sulfur-turf microbial mats were collected from a sulfidic hot spring, Nakabusa spa, Japan. Microscopic observation indicated the microbial community in the sulfur-turf was dominated by a large sausage-shaped bacterium (LSSB) (5–40 µm in cell length) with a large amount of elemental sulfur particles around its cells (Fig. 1). In the 16S rRNA gene clone library analysis, a total of 99 clonal sequences were grouped into nine operational taxonomic units (OTUs) (ST-01 to ST-09) that were affiliated with the five bacterial phyla, *Aquificae* (92% of total clones), *Deinococcus/Thermus* (3%), *Firmicutes* (3%), *Thermodesulfobacteria* (1%) and *Armatimonadetes* (1%) (formerly called the candidate phylum OP10) (Table S1, Fig. S1) [30]. Ninety one clones of ST-B01 and ST-B02 (92% of total clones) showed high sequence similarities with >98% to the LSSB clone (uncultured *Aquificales* bacterium clone NKB2-1) that was previously retrieved from the sulfur-turf microbial mats in the Nakabusa hot spring (Table S1, Fig. S1) [12]. This clearly indicated that the LSSB dominated in the microbial mat obtained in this study.

**Figure 8:**
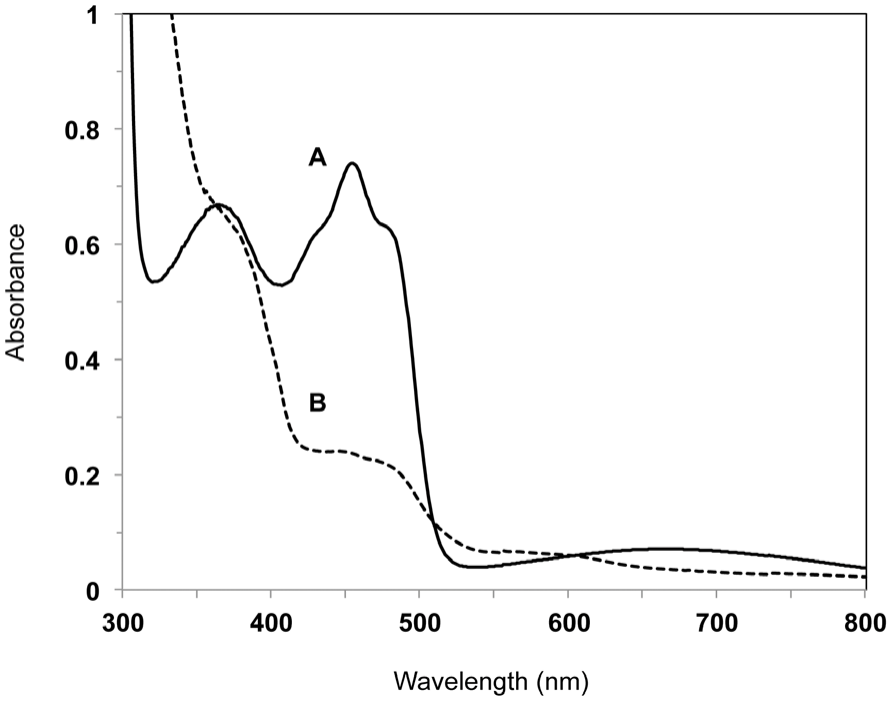
Ultraviolet-visible absorption spectrum of recombinant DhsU1 of the LSSB. DhsU1 (4.5 mg/ml) in 100 mM Tris-HCl buffer (pH 8.0) at room temperature. (A) air-oxidized DhsU1, (B) dithionite-reduced DhsU1.

### Metagenome Sequencing of the LSSB

A fosmid library containing a total of 6,432 clones with average insert size of 36 kbp was constructed using the DNA extracted from the sulfur-turf mats (Table S2). A total of 12,864 reads (approximately 7.5 Mb) were obtained by paired-end sequencing and assembled to 1,720 contigs (Table S2). Based on self-organizing map (first SOM) analysis established by Abe et al. [24], 1,064 of 1,720 contigs were grouped and sorted out as the sequences of LSSB. Further shotgun sequencing of the fosmid clones with the LSSB sequences produced 34,076 reads (approximately 15 Mbp) that generated 3,349 contigs and 10,298 singlets by assembly. We further performed SOM analysis (second SOM) for those contigs and singlets, and eventually obtained a total of 3.7 Mbp sequences (1,504 contigs and 135 singlets) derived from the LSSB genomes. Assuming 1.7 Mbp of genome size of the LSSB based on those of the closest relatives (*Sulfurihydrogenibium azorense* Az-Fu1, 1.6 Mbp; *Sulfurihydrogenibium* sp. strain Y03AOP1, 1.8 Mbp) [31], the expected sequence coverage is approximately×2.2. The sequence completeness of the LSSB was also roughly estimated to be 90% as described by Jones et al. [32] and in the supplementary method (Table S2). The average GC content of the LSSB genome was 34.5% that was similar to those of the known *Sulfurihydrogenibium* isolates (28–35% with the average of 32%) [13]–[17].

### Metagenomic Characterization of Sulfur-oxidation and Carbon Metabolisms in the LSSB

In the Nakabusa hot spring, the main energy sources are the reduced sulfur compounds [11], mainly formed as sulfide that is continuously supplied to the sulfur-turf mats from hot spring water. To verify if the LSSB possesses sulfur oxidation metabolism, we attempted to identify the genes associated with sulfur oxidation in the obtained metagenomic sequences of the organism. Using the BLASTP program, genes encoding the putative sulfur compounds oxidizing enzymes were successfully identified as follows: sulfide dehydrogenase (*dhsU1* and *dhsU2*) (SD) and sulfide-quinone reductase (*sqr1* and *sqr2*) catalyzing the oxidation of sulfide to elemental sulfur; sulfite dehydrogenase (*sor*) mediating further oxidation of sulfite to sulfate; the sulfur oxidation proteins oxidizing thiosulfate to sulfate (*soxX, soxY, soxZ, soxA and soxB*) (Fig. 2 and Table S3). There was no evidence for genes associated with elemental sulfur oxidation. The SD genes formed a cluster with a cytochrome-like gene, a Rieske iron sulfur protein gene, and *sox* genes (Fig. 3). An ORF (no. x0974) encoding flavocytochrome was located in the upstream of the *dhsU1* gene (Fig. 3). An ORF (no. x0976) encoding Rieske iron sulfur protein was displayed between *dhsU1* and *dhsU2* genes (Fig. 3). An ORF (no. x2661) encoding monoheme cytochrome sulfur oxidation protein (*soxX*) was placed in the downstream of the *dhsU2* gene, and the ORFs encoding *sox* genes (*soxY*, *soxZ*, *soxA* and *soxB*) were also located in further downstream of the *soxX* gene (Fig. 3). The following respiration-related genes necessary for sulfide oxidation were also identified: a quinone (no. x1488), cytochrome *c* (no. x0925), cytochrome *bc1* complex (no. x0411 and x0412) and also *cbb3*-type cytochrome *c* oxidase (no. x0212, x0213, x0214 and x0216) as terminal oxidase (Table S3). Those genes were placed in the different locus remote from the sulfur oxidizing genes.

We further identified all the genes required for reductive tricarboxylic acid (rTCA) cycle, which was recently found in the members of the *Aquificae* [33], in the draft genome of LSSB (Table S4). Taken together, our metagenomic analysis clearly suggested that LSSB has the genetic potential to fix carbon dioxide by the rTCA cycle and can grow autotrophically by oxidizing sulfide that is continuously supplied from the hot spring.

### Cloning, Expression, Purification and Enzymatic Assay of Sulfide Dehydrogenase (SD) Gene from LSSB

To biochemically confirm if LSSB can oxidize sulfide, we attempted to clone and express the key gene for sulfide oxidation (putative SD gene) found in the LSSB in this study. Both *dhsU1* and *dhsU2* genes excluding signal peptides were cloned into pET28-b and the heterologous expression system using *E. coli* BL21(DE3) was constructed for both genes. We succeeded in cloning and expression of the *dhsU1* gene, although the *dhsU2* was not expressed possibly due to the *dhsU2* gene sequence harboring two rare codons for the host *E. coli*. We further confirmed the SD activity of the recombinant DhsU1. The SD activity of the crude extract showed 0.031 U/mg (Table 1). The SD activity of the recombinant DhsU1 purified with His-Tag affinity chromatograph showed 0.255 U/mg, indicating 8.3 folds higher activity than that of the crude extract (Table 1). Single band was detected in SDS-PAGE (Fig. 4). The molecular weight was estimated to be 47 kDa consistent with the predicted size based on the deduced amino acid sequence. The enzymatic properties of the recombinant DhsU1 were characterized as described below.

### Amino Acid Sequence Analysis of *dhsU1*

We performed amino acid sequence analysis for a SD gene (*dhsU1*) of the LSSB. Phylogenetic tree constructed using the amino acid sequences of the SD showed that the DhsU1 of the LSSB formed a deep branch with SULAZ_1677 (SD activity has not been reported) derived from *S. azorense* in the phylum *Aquificae* and was distantly related to the other bacterial SDs (Fig. 5). The amino acid sequence similarity between the DhsU1 and SULAZ_1677 was 88%. Comparing to SDs known to have SD activity, the LSSB DhsU1 showed the highest sequence similarity (50%) to flavocytochrome *c* sulfide dehydrogenase (Fcsd) flavoprotein subunit (FccB) from *Allochromatium vinosum* within the *Gammaproteobacteria*.

Multiple amino acid sequence alignment of the DhsU1 of the LSSB and other bacterial SDs known to have SD activity showed the conserved amino acid residues, i.e. Cys53 residue at FAD binding site, Cys173 and Cys344 residues in forming a disulfide bridge, and Glu169 residue at the active site (Fig. 6). All the above residues were previously identified with the X-ray crystal structure analysis in a well-studied SD, Fcsd from *A. vinosum* [34].

Using SignalP search, signal sequences were found in the N-terminal within the first 26 amino acid sequence of the DhsU1 of the LSSB (data not shown), and were related to the sequence of the twin-arginine translocation (Tat) peptide. Tat peptide was used for secretion of various proteins into the periplasmic space [35], suggesting that the DhsU1 was localized in the periplasm of the cell.

### Enzymatic Characteristics of Sulfide Dehydrogenase (DhsU1)

The optimal temperature and pH of the purified recombinant DhsU1 were 60°C and 8.0, respectively (Fig. 7A, B). In thermostability test, DhsU1 could be tolerant high temperature and showed 86% and 30% of the residual SD activities when the enzyme was preheated for 30 min at 70°C and 80°C, respectively (Fig. 7C). The SD activity of the recombinant DhsU1 was not observed when elemental sulfur, sulfite and thiosulfate were used as the substrate. The effect of ferricyanide, an inhibitor of SD [36]–[38], on the DhsU1 activity was investigated. With the addition of 10 µM ferricyanide, the residual activity decreased to 24.5% of the control (without inhibitor). The addition of 25 µM ferricyanide completely inhibited the activity (data not shown). The *K*_m_ values of the DhsU1 for sulfide and horse heart cytochrome *c* were 16.7 µM and 52.0 µM, respectively. The DhsU1 had a *V*_max_ value of 2.5 µmol/mg^−1^/min^−1^ and a *K*_cat_ value of 1.9 s^−1^ (Table 2).

UV-visible spectrum analysis was performed for the DhsU1, since the deduced amino acid sequence of the DhsU1 possessed a FAD binding domain and the macroscopic color of the purified recombinant DhaU1 was yellow. The DhsU1 had diagnostic peeks at 364 nm and 454 nm (Fig. 8), and these peeks disappeared after addition of dithionite (Fig. 8).

## Discussion

The sulfur-turf microbial mat developed in the high-temperature sulfidic hot spring forms a unique ecosystem where hydrogen sulfide is the primary energy source and constantly supplied [11], [39]. However, microbial functions associated with sulfur cycle in the sulfur-turf mats are not fully understood mainly due to the difficulty in cultivation of the main constituents. The large sausage-shaped bacterium (LSSB) was predominant in the sulfur-turf mats, and was yet to be cultured. Several previous reports suggest that the organism may be a chemolithoautotrophic and microaerophilic sulfide oxidizing thermophile [2], [19], [40], [41]. However, there is no direct evidence showing whether or not the organism possesses the responsible genes and enzymes for sulfide oxidation due to the absence of an isolated strain. To verify this, we conducted metagenomic and enzymatic analyses for the sulfur-turf microbial mats focusing on numerically abundant LSSB.

Using metagenomic approach, we obtained approximately 3.7 Mbp of LSSB sequences (Table S2) covering approximately 90% of its genome. This allowed us to analyze the metabolic potential of the LSSB. 1,472 ORFs were found out from the LSSB draft genome sequences. From those ORFs, all the necessary genes for sulfide oxidation such as sulfide dehydrogenase (SD) gene, sulfide-quinone reductase (Sqr) gene, and other respiration-related genes for quinone synthesis, cytochrome, cytochrome *bc1* complex and terminal oxidase were identified in the present study (Fig. 2 and Table S3). In addition, we found all the genes required for reductive tricarboxylic acid (rTCA) cycle for carbon dioxide fixation in the LSSB (Table S4). The findings clearly suggested that the LSSB is an autotrophic sulfide oxidizer that can fix carbon using rTCA cycle and obtain energy by sulfide oxidation. The genes responsible for sulfite and thiosulfate oxidations (e.g. *sor, soxX, soxY, soxZ, soxA* and *soxB*) were also found in the ORFs, suggesting that the organism have an ability to oxidize sulfite and thiosulfate as well (Table S3). However, the observation of abundant elemental sulfur particles on the LSSB cells suggests that sulfide oxidation can serve as a main energy-yielding pathway. The terminal oxidase identified in the genome was the *cbb3*-type supporting microaerobic respiration (Table S3), and no aerophilic-type terminal oxidase such as *aa3*-type has been found in the genome fragments. The LSSB may therefore oxidize sulfide under microaerobic conditions as suggested in the previous report [12], [41]. Such ability to autotrophically oxidize sulfide under microaerobic conditions is advantageous in the sulfidic high-temperature hot spring, and provides the LSSB a distinct niche as a primary producer in such ecosystem.

Recently, based on a metagenomic analysis, Inskeep et al. (2010) also reported that filamentous *Aquificae* bacteria were dominating in a white streamer in sulfidic hot spring water at Mammoth Hot Spring site in Yellowstone National Park [42]. Indeed, like our study, majority (∼90%) of the metagenomic sequences exhibits high similarity (>90% of nucleotide identity) to the genome of *Sulfurihydrogenibium* sp. Y03AOP1 within the *Aquificae* [31], and contains genes responsible for inorganic sulfur compound oxidation, respiration, and carbon fixation via rTCA cycle, although no physiological or biochemical evidence of the metabolisms is not shown. Such similar findings in the two geographically distinct hot springs, Mammoth Hot Spring (USA) and Nakabusa spring (Japan), suggest the importance of the dominant *Aquificae* bacterium and its sulfide oxidation metabolism in the sulfidic geothermal environments worldwide.

Sulfide dehydrogenase (SD) is a key enzyme for sulfide oxidation and known to be a flavoprotein that oxidizes sulfide to elemental sulfur and transfers electrons to cytochrome. In this study, we succeeded in the cloning and heterologous expression of the SD gene (*dhsU1*) found in the genome fragments of the uncultured LSSB (Fig. 4, Table 1). In fact, the crude and affinity purified DhsU1 showed the significant SD activity. The SD activity was completely inhibited by a known inhibitor, ferricyanide, [36]–[38], also supporting that the recombinant DhsU1 is SD. This is the first report showing the successful isolation and heterologous expression of the sulfide oxidizing gene by using the metagenome obtained from the hot spring microbial mats. Moreover, this also provides the first evidence that the member of the phylum *Aquificae* possessed SD activity. SD-like sequences have been identified in the genomes of several *Aquificae* bacteria isolated from hot springs, which suggests that SD can play an important role in their sulfide oxidation in the environments.

The enzymatic properties of the recombinant DhsU1 derived from the LSSB were further characterized. The DhsU1 showed high substrate specificity toward sulfide and its SD activity was not detected using elemental sulfur, sulfite or thiosulfate. The optimal temperature and pH of the recombinant DhsU1 were consistent with the environmental conditions (water temperature, 55–70°C; pH, 7.3–8.3) in the sulfur-turf microbial ecosystem. The *K*_m_ of the DhsU1 for sulfide (16.5 µM) was eight times higher than those of the previously known bacterial SDs [38], [43], suggesting that the enzyme has relatively low affinity to sulfide. This also fits to the environmental conditions where relatively high concentration of sulfide is continuously supplied [11]. These findings indicate that the SD from the LSSB adapted to the inhabiting environment.

Amino acid sequence analysis of the previously known SDs indicated that the DhsU1 of the LSSB was most similar (50% of sequence similarity) to the flavocytochrome *c* sulfide dehydrogenase (Fcsd) flavoprotein subunit of (FccB) from *Allochromatium vinosum* within the *Gammaproteobacteria* [34]. Both enzymes harbor a signal peptide, the twin-arginine translocation (Tat) peptide, indicating those are located in the periplasmic space. The amino acid sequences of the DhsU1 of the LSSB had a flavin adenine dinucleotide (FAD) binding site at the same position of Cys42 as the Fcsd from *A. vinosum*, indicating that FAD is covalently bound to the DhsU1 (Fig. 6). The identical residues of Cys161 and Cys337 in the Fcsd, which are considered as forming disulfide bond, were also found in the DhsU1 (Fig. 6). The previous report [34] indicated that the disulfide bond is adjacent to the FAD. Griesbeck et al. (2002) also identified the pair of cysteines in the Sqr from *Rhodobacter capsulatus* and found that the SD activity disappeared when respective cysteine residue was replaced with serine [44]. The fact that those cysteine residues are conserved among the SDs from the LSSB (the phylum *Aquificae*) and *A. vinosum* (the *Gammaproteobacteria*) and Sqr from *R. capsulatus* (the *Alphaproteobacteria*), despite their distinct placement in phylogeny, strongly suggests the importance of the pair residues for maintaining their SD activities.

The spectrum analysis of the protein showed two diagnostic peaks of flavin at 364 and 454 nm without other conspicuous peaks (Fig. 8). Those peaks were decreased by addition of dithionite, indicating that flavin was completely reduced (Fig. 8). Furthermore, SDS-PAGE showed a single band (Fig. 4) and gel filtration indicated the purified recombinant DhsU1 is a monomer (data not shown). These results show that the purified recombinant DhsU1 is a monomeric flavoprotein. So far, the monomeric flavoprotein SD has been observed only in two species, *Paracoccus pantotrophus* (the phylum *Proteobacteria*) and *Chlorobaculum tepidum* (the phylum *Chlorobi*) [43], [45]. All the other SDs is the heterodimer protein composed of flavoprotein subunit and di-heme or mono-heme cytochrome subunit [38], [46]–[48]. Although the DhsU1 amino acid sequence showed high similarity to the heterodimer SD from *A. vinosum*, the DhsU1 structure was more similar to the monomer SD from *P. pantotrophus* and *C. tepidum*. Taken together with the fact that LSSB belongs to the phylum *Aquificae*, an early branching lineage in the domain *Bacteria* in the 16S rRNA gene-based phylogeny, the findings suggest that the monomer-type SD may be an ancient origin of the bacterial SD. This will provide further insight into the largely unknown molecular evolution of the enzyme. Such enzymatic information could not be obtained by using only metagenome data but by a combined use of metagenomic and biochemical approaches in this study.

In conclusion, this study using metagenomic approach strongly suggested that the uncultured LSSB dominated in the high-temperature sulfidic hot spring possessed autotrophic sulfide oxidizing metabolism and could grow as a main primary producer in such ecosystem. Based on the metagenomic information, we succeeded in cloning and heterologous expression of the SD of the LSSB, a critical enzyme for oxidation of sulfide that is considered as the primary electron donor in its habitat. The enzymatic properties, i.e., its thermophily, moderately alkalophile and a high *K*_m_ for sulfide, were consistent with the environmental conditions, indicating that the SD of the LSSB had adapted to the sulfidic hot spring environment. This is the first report showing that the LSSB has the ability to oxidize sulfide using the unique monomeric SD in the hot spring environment.

## Supporting Information

Figure S1 **The phylogenetic composition of the microbial community in the sulfur-turf mat based on 16S rRNA gene clone library analysis.**
(TIF)

Table S1 **Phylogenetic affiliations and numbers of bacterial 16S rRNA gene sequences in the clone library generated from the sulfur-turf microbial mats.**
(DOCX)

Table S2 **Summary statistics for the sulfur-turf metagenome sequencing and assembling, and the draft genome of the LSSB.**
(DOCX)

Table S3 **Sulfur oxidation related genes identified from the draft genome of the LSSB.**
(DOCX)

Table S4 **CO_2_ fixation related genes identified from the draft genome of the LSSB.**
(DOCX)

Method S1 **Genome sequence completeness estimation.**
(DOCX)
