# Supplementary figures and images for: Metagenomic and Biochemical Characterizations of Sulfur Oxidation Metabolism in Uncultured Large Sausage-Shaped Bacterium in Hot Spring Microbial Mats
Source: PLoS One. 2012 Nov 21;7(11):e49793. doi: 10.1371/journal.pone.0049793 (PMC3504083; doi:10.1371/journal.pone.0049793)

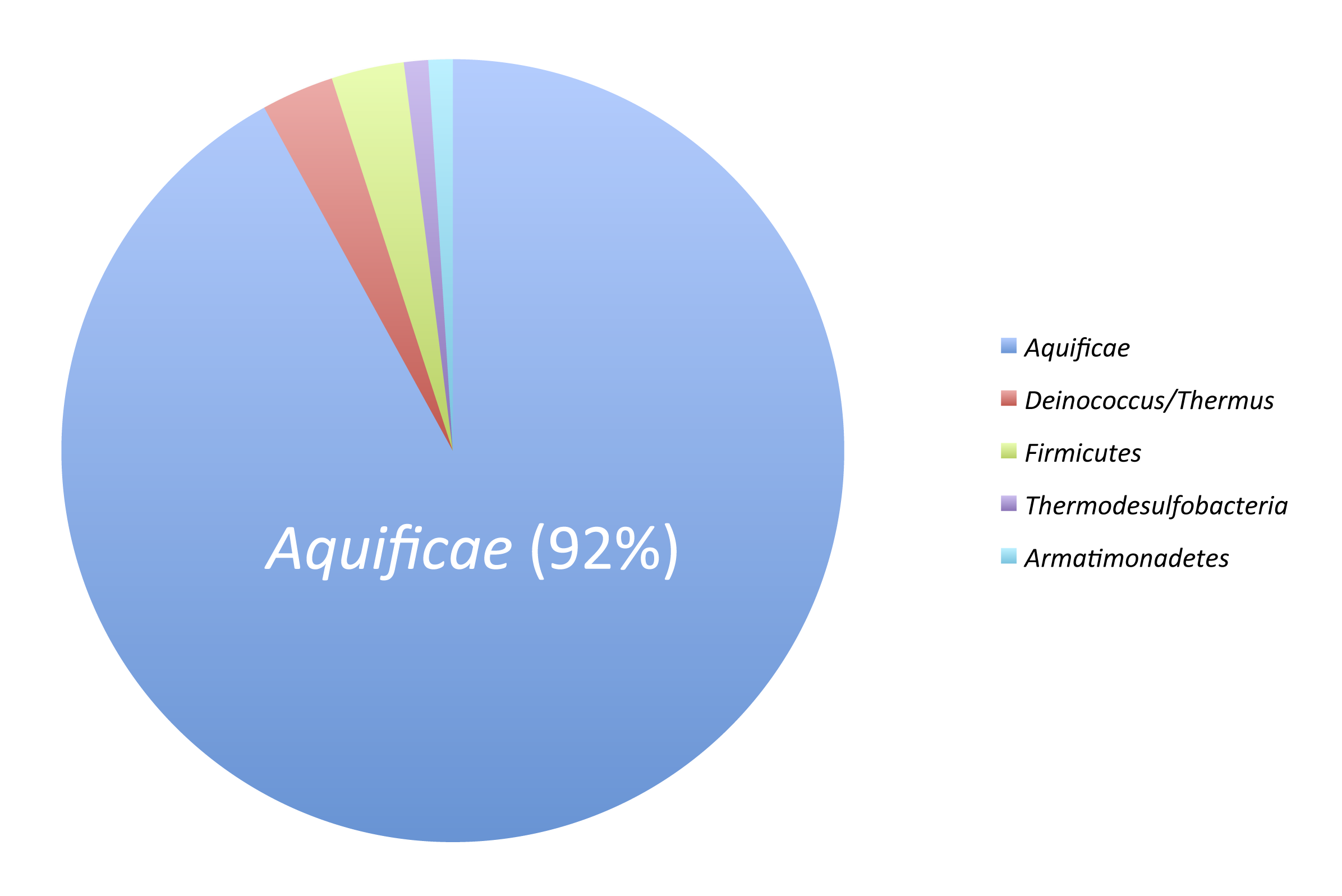

Supplement: Figure S1 — The phylogenetic composition of the microbial community in the sulfur-turf mat based on 16S rRNA gene clone library analysis. (TIF) [file pone.0049793.s001.tif]
