# Supplementary material for: Metagenomic and Biochemical Characterizations of Sulfur Oxidation Metabolism in Uncultured Large Sausage-Shaped Bacterium in Hot Spring Microbial Mats
Source: PLoS One. 2012 Nov 21;7(11):e49793. doi: 10.1371/journal.pone.0049793 (PMC3504083; doi:10.1371/journal.pone.0049793)
Supplement: Table S1 — Phylogenetic affiliations and numbers of bacterial 16S rRNA gene sequences in the clone library generated from the sulfur-turf microbial mats. (DOCX) [file pone.0049793.s002.docx]

Table S1. Phylogenetic affiliations and numbers of bacterial 16S rRNA gene sequences in the clone library generated from the sulfur-turf microbial mats.

| OTU | No. of clones | Phylogenetic group (phylum) | Closest relative | Accession no. | Similarity (%) |
| --- | --- | --- | --- | --- | --- |
| ST-B01 | 87 | *Aquificae* | Uncultured *Aquificales* bacterium clone NKB2-1 | FR691794 | 99 |
| ST-B02 | 4 | *Aquificae* | Uncultured *Aquificales* bacterium clone NKB2-1 | FR691794 | 98 |
| ST-B03 | 2 | *Thermus* | Uncultured bacterium clone NKB_58_047 | JF826980 | 98-99 |
| ST-B04 | 1 | *Thermus* | Uncultured *Thermus* sp. clone VrW-70 | FM994921 | 99 |
| ST-B05 | 1 | *Firmicutes* | *Aneurinibacillus* sp. NBRC 15378 | AB680851 | 99 |
| ST-B06 | 1 | *Firmicutes* | Uncultured bacterium clone E1-15 | EF600594 | 98 |
| ST-B07 | 1 | *Firmicutes* | Uncultured bacterium clone E1-15 | EF600594 | 97 |
| ST-B08 | 1 | *Thermodesulfobacteria* | Uncultured bacterium clone NKB_63_56 | JF826988 | 99 |
| ST-B09 | 1 | *Armatimonadetes* (formerly OP10) | Unnamed isolate S2R-194 | FN545892 | 100 |
