# Supplementary material for: Metagenomic and Biochemical Characterizations of Sulfur Oxidation Metabolism in Uncultured Large Sausage-Shaped Bacterium in Hot Spring Microbial Mats
Source: PLoS One. 2012 Nov 21;7(11):e49793. doi: 10.1371/journal.pone.0049793 (PMC3504083; doi:10.1371/journal.pone.0049793)
Supplement: Table S2 — Summary statistics for the sulfur-turf metagenome sequencing and assembling, and the draft genome of the LSSB. (DOCX) [file pone.0049793.s003.docx]

Table S2. Summary statistics for the sulfur-turf metagenome sequencing and assembling, and the draft genome of the LSSB.

| Fosmid information: |  |
| --- | --- |
| Number of fosmid clones | 6,432 |
| Average of insert length | 36 kbp |
| Paired-end sequencing information: |  |
| Paired-end sequenced fosmids | 12,864 |
| Average of read length | 579 bp^a^ |
| Number of contigs | 1,720 (2.4 Mbp) |
| First SOM analysis information: |  |
| Applied contigs | 1,720 |
| Applied reference complete genomes | 212 species |
| Number of grouped contigs | 1,064 |
| Shotgun sequencing information: |  |
| Shotgun-sequenced reads | 34,076 |
| Number of contigs | 3,349 (6.0 Mbp) |
| Number of singles | 10,296 (7.0 Mbp) |
| Second SOM analysis information: |  |
| Applied contigs | 3,349 |
| Applied singlets | 10,296 |
| Applied reference complete genomes | 212 species |
| Number of grouped contigs | 1,504 |
| Draft genome information of LSSB: |  |
| Total sequence length | 3.7 Mbp |
| Number of contigs | 1,504 |
| Total contigs size | 3.6 Mbp |
| Number of singlets | 135 |
| Total singlets size | 0.1 Mbp |
| Average of insert G+C content | 34.5% |
| Genome size | 1.7 Mbp^b^ |
| Genome completeness | 90%^b^ |
| Number of predicted ORFs | 1,472 |

a: >20 Phred quality score, b: the coverage was estimated from a ratio of core genes and tRNA synthetase genes.
