# Supplementary material for: Metagenomic and Biochemical Characterizations of Sulfur Oxidation Metabolism in Uncultured Large Sausage-Shaped Bacterium in Hot Spring Microbial Mats
Source: PLoS One. 2012 Nov 21;7(11):e49793. doi: 10.1371/journal.pone.0049793 (PMC3504083; doi:10.1371/journal.pone.0049793)
Supplement: Table S3 — Sulfur oxidation related genes identified from the draft genome of the LSSB. (DOCX) [file pone.0049793.s004.docx]

Table S3. Sulfur oxidation related genes identified from the draft genome of the LSSB.

| ORF no. | Gene name | Putative product | Identity (%) | Organism | Accession no. |
| --- | --- | --- | --- | --- | --- |
| Sulfide oxidation | |  |  |  |  |
| x0975 | *dhsU1* | Sulfide dehydrogenase | 88 | *Sulfurihydrogenibium azorense* Az-Fu1 | YP_002729635 |
| x0977 | *dhsU2* | Sulfide dehydrogenase | 89 | *Sulfurihydrogenibium azorense* Az-Fu1 | YP_002729637 |
| x0294 | *sqr1* | Sulfide-quinone reductase | 96 | *Sulfurihydrogenibium azorense* Az-Fu1 | YP_002728909 |
| x2263 | *sqr2* | Sulfide-quinone reductase | 88 | *Sulfurihydrogenibium azorense* Az-Fu1 | YP_002728108 |
| Sulfite oxidation | | | |  |  |
| x1152 | *sor* | Sulfite dehydrogenase | 88 | *Sulfurihydrogenibium azorense* Az-Fu1 | YP_002728583 |
| Sox complex | |  |  |  |  |
| x2661 | *soxX* | Monoheme cytochrome | 81 | *Sulfurihydrogenibium azorense* Az-Fu1 | YP_002729638 |
| x1541 | *soxY* | Sulfur oxidation protein | 79 | *Sulfurihydrogenibium* sp. YO3AOP1 | YP_001930710 |
| x2376 | *soxZ* | Sulfur oxidation protein | 88 | *Sulfurihydrogenibium azorense* Az-Fu1 | YP_002729641 |
| x1543 | *soxA* | Diheme cytochrome | 85 | *Sulfurihydrogenibium azorense* Az-Fu1 | YP_002729642 |
| x1142 | *soxB* | Sulfur oxidation protein | 93 | *Sulfurihydrogenibium azorense* Az-Fu1 | YP_002729643 |
| Sulfur oxidation and respiration related | | | |  |  |
| x0974 | - | Flavocytochrome | 79 | *Sulfurihydrogenibium azorense* Az-Fu1 | YP_002729634 |
| x0925 | - | Cytochrome *c*552 | 54 | *Sulfurihydrogenibium* sp. YO3AOP1 | YP_001931731 |
| x1488 | - | Quinone | 72 | *Sulfurihydrogenibium azorense* Az-Fu1 | YP_002728668 |
| x0411 | - | Cytochrome B | 96 | *Sulfurihydrogenibium azorense* Az-Fu1 | YP_002728284 |

| x0412 | - | Ubiquinol cytochrome *c* oxidoreductase cytochrome *c*1 subunit | 91 | *Sulfurihydrogenibium azorense* Az-Fu1 | YP_002728285 |
| --- | --- | --- | --- | --- | --- |
| x0976 | *qcrA* | rieske-I iron sulfur protein | 80 | *Sulfurihydrogenibium azorense* Az-Fu1 | YP_002729636 |
| x0212 | - | Cytochrome *c* | 95 | *Sulfurihydrogenibium azorense* Az-Fu1 | YP_002728687 |
| x0213 | - | Cytochrome *c* oxidase subunit I | 98 | *Sulfurihydrogenibium azorense* Az-Fu1 | YP_002728688 |
| x0214 | - | Cytochrome *c* oxidase *Cbb*-3 type, subunit II | 96 | *Sulfurihydrogenibium azorense* Az-Fu1 | YP_002728689 |
| x0216 | - | Cytochrome *c* oxidase *Cbb*-3 type, subunit III | 96 | *Sulfurihydrogenibium azorense* Az-Fu1 | YP_002728692 |

*Continued*
