# Supplementary material for: Metagenomic and Biochemical Characterizations of Sulfur Oxidation Metabolism in Uncultured Large Sausage-Shaped Bacterium in Hot Spring Microbial Mats
Source: PLoS One. 2012 Nov 21;7(11):e49793. doi: 10.1371/journal.pone.0049793 (PMC3504083; doi:10.1371/journal.pone.0049793)
Supplement: Table S4 — CO2 fixation related genes identified from the draft genome of the LSSB. (DOCX) [file pone.0049793.s005.docx]

Table S4. CO_2_ fixation related genes identified from the draft genome of the LSSB.

| ORF no. | Gene name | Putative product | Identity (%) | Organism | Accession no. |
| --- | --- | --- | --- | --- | --- |
| x1551 | *aclA* | ATP citrate lyase | 94 | *Sulfurihydrogenibium* sp. YO3AOP1 | YP_001931228 |
| x0599 | *aclB* | ATP citrate lyase | 97 | *Sulfurihydrogenibium azorense* Az-Fu1 | YP_002728518 |
| x0311 | *mdh* | malate dehydrogenase | 96 | *Sulfurihydrogenibium azorense* Az-Fu1 | YP_002728619 |
| x0312 | *fumA* | fumarate hydratase | 94 | *Sulfurihydrogenibium azorense* Az-Fu1 | YP_002728620 |
| x0313 | *fumB* | fumarate hydratase, class I | 97 | *Sulfurihydrogenibium azorense* Az-Fu1 | YP_002728622 |
| x1291 | *frd* | fumarate reductase, iron-sulfur subunit | 95 | *Sulfurihydrogenibium azorense* Az-Fu1 | YP_002728121 |
| x0315 | *sucC* | succinyl-CoA synthetase subunit beta | 98 | *Sulfurihydrogenibium azorense* Az-Fu1 | YP_002728623 |
| x0318 | *sucD* | succinyl-CoA synthetase subunit alpha | 97 | *Sulfurihydrogenibium* sp. YO3AOP1 | YP_001930810 |
| x0320 | *forA* | subunit of 2-oxoglutarate:ferredoxin oxidoreductase | 94 | *Sulfurihydrogenibium azorense* Az-Fu1 | YP_002728627 |
| x0322 | *forG* | subunit of 2-oxoglutarate:ferredoxin oxidoreductase | 95 | *Sulfurihydrogenibium azorense* Az-Fu1 | YP_002728629 |
| x0321 | *forB* | subunit of 2-oxoglutarate:ferredoxin oxidoreductase | 93 | *Sulfurihydrogenibium azorense* Az-Fu1 | YP_002728628 |
| x0319 | *forD* | subunit of 2-oxoglutarate:ferredoxin oxidoreductase | 92 | *Sulfurihydrogenibium azorense* Az-Fu1 | YP_002728626 |
| x0600 | *icd* | isocitrate dehydrogenase | 89 | *Sulfurihydrogenibium azorense* Az-Fu1 | YP_002728517 |
| x0568 | *acnB* | bifunctional aconitate hydratase 2/2-methylisocitrate dehydratase | 96 | *Sulfurihydrogenibium azorense* Az-Fu1 | YP_002729479 |
