# Supplementary material for: Metagenomic and Biochemical Characterizations of Sulfur Oxidation Metabolism in Uncultured Large Sausage-Shaped Bacterium in Hot Spring Microbial Mats
Source: PLoS One. 2012 Nov 21;7(11):e49793. doi: 10.1371/journal.pone.0049793 (PMC3504083; doi:10.1371/journal.pone.0049793)
Supplement: Method S1 — Genome sequence completeness estimation. (DOCX) [file pone.0049793.s006.docx]

**Supplementary method**

**Genome sequence completeness estimation.** The large sausage-shaped bacterium (LSSB) genome sequence completeness was estimated by two different methods. First, the presence or absence of the 109 core genes present in the genomes of the closest relatives (*Sulfurihydrogenibium azorense* Az-Fu1, *Sulfurihydrogenibium* sp. strain Y03AOP1) [[1](#_ENREF_1)] was searched in the LSSB draft genome. Consequently, the 98 core genes were detected in the LSSB genome, indicating that the genome sequence completeness is approximately 90%. Secondly, aminoacyl transfer RNA (tRNA) synthetases in the LSSB genome were identified for 18 of 20 amino acids, also suggesting that the completeness of the LSSB genome was around 90%.

**References**

1. Reysenbach AL, Hamamura N, Podar M, Griffiths E, Ferreira S, et al. (2009) Complete and draft genome sequences of six members of the Aquificales. J Bacteriol 191: 1992-1993.
